# Obesity, body weight stigma and biomedical oversimplification: moving beyond BMI

**DOI:** 10.1093/eurpub/ckac129.355

**Published:** 2022-10-25

**Authors:** S Stranges

**Affiliations:** Department of Epidemiology and Biostatistics, Western University, London, Canada

## Abstract

Body Mass Index (BMI) is one of the most important factors considered to analyse health status. High BMI scores are usually associated with physical or mental disorders, such as cardiovascular disease, diabetes, inflammation, dyslipidemia and depression. However, obesity is usually characterised by the presence of various comorbidities that should be addressed as part of a unique, more complicated picture. Emerging evidence suggests that a deeper acknowledgment of the complexity of obesity as disease, including its comorbidities, should take place in the near future. Overweight, obesity and related disorders have been associated with direct healthcare-related costs and the indirect costs of productivity loss, mostly due to the presence of comorbidities rather than to body weight in general. The belief that weight loss is the best recommendation for people with high BMI as the only path to health could be questioned. In fact, it would seem that weight loss in some subjects does not necessarily coincide with any health improvement. Thus, the relationship between weight status and health expenditure is complex with particular reference to the fact that costs of disease are higher in high-comorbid profiles independently of weight status. Over the last years, researchers have also studied some conditions, such as metabolically healthy overweight and obesity. Recent studies deal with alternatives to conventional weight-loss approaches that could be more effective in health terms. Conventional methods of weight loss are based on calorie restriction and increased energy expenditure instead of unconventional methods, such as the weight-neutral program, which is based on the crucial concept of “mindfulness” to underline the importance of intuitive eating, self-care, pleasurable exercise and size-acceptance. The health benefits related to such types of approaches include physical, psychological and behavioural improvements.

